# Training communication skills in a multiuser medical virtual reality simulation: a qualitative, observational study

**DOI:** 10.1186/s41077-025-00386-8

**Published:** 2025-11-24

**Authors:** Lotte Cools, Rani Van Schoors, Fien Depaepe, Eline Dancet, Nicolas Delvaux

**Affiliations:** 1 Department of Public Health and Primary Care, KU Leuven, Etienne Sabbelaan 53 bus 7700, Kortrijk, Belgium; 2 Centre of Instructional Psychology and Technology, KU Leuven, Dekenstraat 2, Leuven, Belgium; 3 Itec, Research Group of Imec and KU Leuven, Etienne Sabbelaan 51, Kortrijk, Belgium; 4 Department of Public Health and Primary Care, KU Leuven, Kapucijnenvoer 7 blok g bus 7001, Leuven, Belgium; 5 Interfaculty Centre for Biomedical Ethics and Law, KU Leuven, Kapucijnenvoer 7 blok g bus 7001, Leuven, Belgium

**Keywords:** Undergraduates, Interpersonal communication, Intraprofessional communication, Core communication skills

## Abstract

**Background:**

Simulation-based education is a well-established training technique in medical curricula, also for communication skills. Virtual reality (VR) technology can enhance this form of experience-based learning. How VR interacts with training communication skills for interpersonal and interprofessional medical encounters is, however, unclear. This study investigates how VR influences communication skills and behaviors in patient-student and team encounters in medical undergraduate simulations, in order to make recommendations for VR simulation-based communication skills training (CST).

**Methods:**

We conducted a study with 22 third-year medical students completing a dyadic VR simulation (Smart Collaboration Tutor software). We coded communication skills and behaviors for team and patient-student communication in videorecorded VR simulations. We then analyzed communication patterns and finally developed themes for VR-mediated CST.

**Results:**

Our findings revealed that students preferred the core communication skill of asking questions, informing, and thinking aloud as process communication skills in a VR simulation. Nonverbal and paraverbal behaviors were used with unclear intent. VR negatively impacted the focus of attention and flow of simulation-based communication skills training.

**Discussion:**

Dyadic VR simulations tend to emphasize team and task-oriented communication. Its value for patient-student and relation-oriented communication is unclear. VR influenced conversational turn-taking by altering visual and auditory perceptions. Cognitive load was enhanced, potentially diverting attention from communication goals and observational focus.

**Conclusion:**

Multiuser VR simulation shows certain possibilities for CST in medical undergraduate simulations. Recommendations on the contextual design of VR simulations, however, need to be taken into account to safeguard the focus of attention and flow of CST.

**Supplementary Information:**

The online version contains supplementary material available at 10.1186/s41077-025-00386-8.

## Background

Communication skills are central to any clinical encounter [1, 2]. Communication skills training (CST), targeting interpersonal and interprofessional communication, is key to undergraduate medical curricula [3–5]. During medical training, students are immersed in a multitude of clinical and educational encounters with simulated and real patients, team members (peers), and supervisors/facilitators [6]. Learning to apply verbal, paraverbal, and nonverbal communication skills efficiently and authentically within these encounters is challenging.

Simulation-based education (SBE) proved to be instrumental in acquiring communication skills, as it facilitates experience-based learning which is core to medical education [3–5]. Simulation involves creating a situation or environment in which participants experience a representation of a real medical event. A complete medical-educational simulation activity consists of three phases: prebriefing, simulation, and debriefing [6]. These simulation activities facilitate communication skill acquisition in a safe environment, due to the affordances of feedback, repetition, and adaptability of difficulty levels [7–9]. However, sustainably organizing these high-quality, collaborative experience-based learning activities is challenging because of accessibility and organizational issues (e.g., faculty training, material resources, and financial reasons) [10].

Extended reality (XR) technologies can meet these challenges through immersive, high-quality SBE [4, 11–13], with virtual reality (VR) offering full immersion in a virtual environment, including possible interaction. Studies comparing learning outcomes (e.g., knowledge, communication team training) for VR and traditional simulations show the potential of VR [14–16]. Qualitative studies on simulation contexts mostly focus on experiences and general communication [17, 18]. Further investigation is therefore needed to gain understanding of how it influences students’ acquisition of specific communication skills [19, 20].

This study aimed to investigate simulation-based communication skills training (SB-CST) in the context of multiuser VR of undergraduate medical education, focused on specific communication skills. We investigated how VR influenced communication skills and behaviors in patient-student and team encounters in medical undergraduate simulations, in order to make recommendations for VR SB-CST.

## Methods

### Study design

We performed a qualitative, observational study to explore and analyze students’ communication skills and behaviors during a dyadic VR simulation activity. The study was conducted by a multidisciplinary research team with medical, educational, and communication expertise. We used Standards for Reporting Qualitative Research (SRQR) [21] to report our findings.

### Study setting

**Fig. 1:**
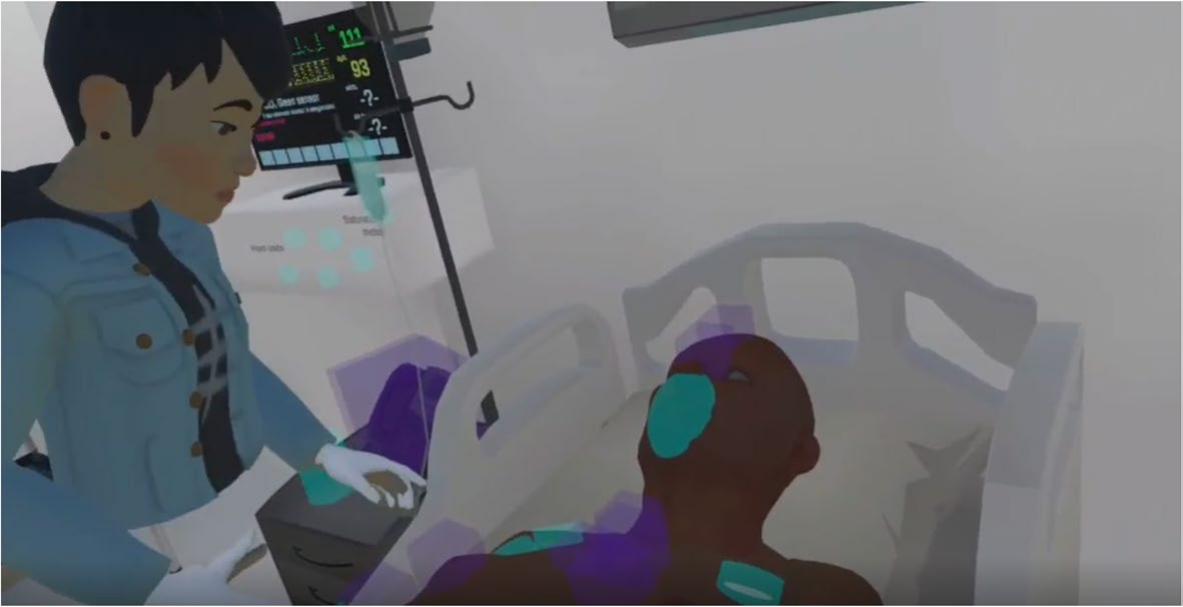
Student- and patient-avatar in the SCOT VR environment [22]

We used the Smart Collaboration Tutor software (SCOT) [22] and Meta Quest Pro hardware [23] (i.e., hand controllers, head-mounted displays (HMD) with muted built-in audio) to practice a semi-acute, bedside scenario in immersive, interactive VR. SCOT is not publicly available. It is designed to encourage students’ patient-related communication (e.g., parameters visible to the examining student), with the patient remaining communicative during the scenario. Student-avatars displayed body movement, dynamic facial expressions, and eye contact via facial and eye recognition technology. Patient-avatars were static, with one neutral facial expression, without eye contact or body movement. One teacher facilitated the simulation, observing student-avatars on a monitor. Teacher-facilitators’ (TF) tasks included dashboard operation, voicing scripted (e.g., patient) and unscripted non-visualized contacts (e.g., physician/lab) and providing written nudges (guiding cues) (Fig. 1). The TF ended simulations after 15 min, followed by a live debriefing. During simulation activities, two researchers of the multidisciplinary team were present for observation and technological support.

### Participants and recruitment

Recruitment was conducted via the university’s digital platform, targeting third-year students in the Bachelor of Medicine. Students registered and received an invitation for the pretraining 3 weeks prior to the simulation [24]. The 15-min pretraining familiarized students with the VR environment and hardware. Absent students received pretraining at the start of the simulation. We recruited one TF experienced with SCOT. We obtained participants’ informed consents before the simulation, during which students were informed of the study’s communication focus. The number of registered students (*N* = 22) determined the number of dyadic simulation activities (*N* = 11), organized in April and May 2024.

### Data collection

**Fig. 2:**
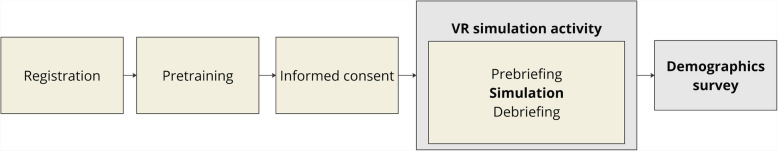
Study stages and data collection procedure (with points of data collection in bold)

We collected qualitative data during and after VR simulation activities [6], following a uniform procedure (Fig. 2).

**Fig. 3:**
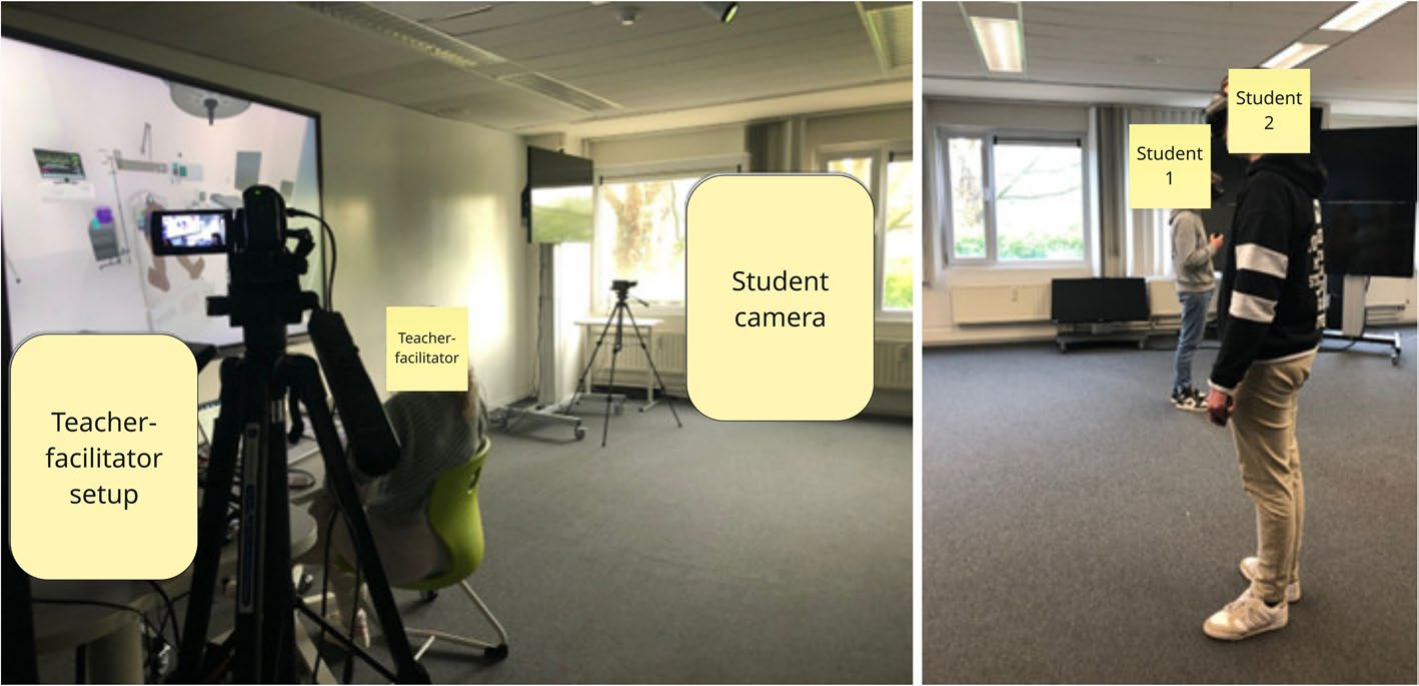
Teacher-facilitator setup, positioning of student-pair, and camera setup for VR simulation

We observed students’ communication skills and behaviors in simulations while taking field notes and through videorecorded simulations for further analysis (Fig. 3). Afterwards, we collected participants’ demographics (e.g., age, sex, number of VR experiences) through an online survey.

### Data analysis

Demographic data were summarized. We pseudonymized data at the transcription phase, with participant codes only retraceable by the primary researcher. This researcher transcribed simulations verbatim and added transcription notations in line with a conversation analysis approach for transcribing nonverbal and paraverbal behaviors observed during simulations (e.g., silences, interruptions) (Appendix 1 ) [25]. We used NVivo 14 software to analyze data [26].

We performed thematic analysis with a hybrid coding approach to identify and explore communication skills and behaviors in VR simulation [27, 28]. First, we developed a coding manual. A deductive coding approach was used to identify predefined communication skills in patient-student and dyadic team communication orientation. In addition, an inductive coding approach was used to explore communication orientations and behaviors that coexisted with the deductive skill set. We focused on verbal communication; however, we included a selection of paraverbal and nonverbal skills/behaviors based on observations.

**Fig. 4:**
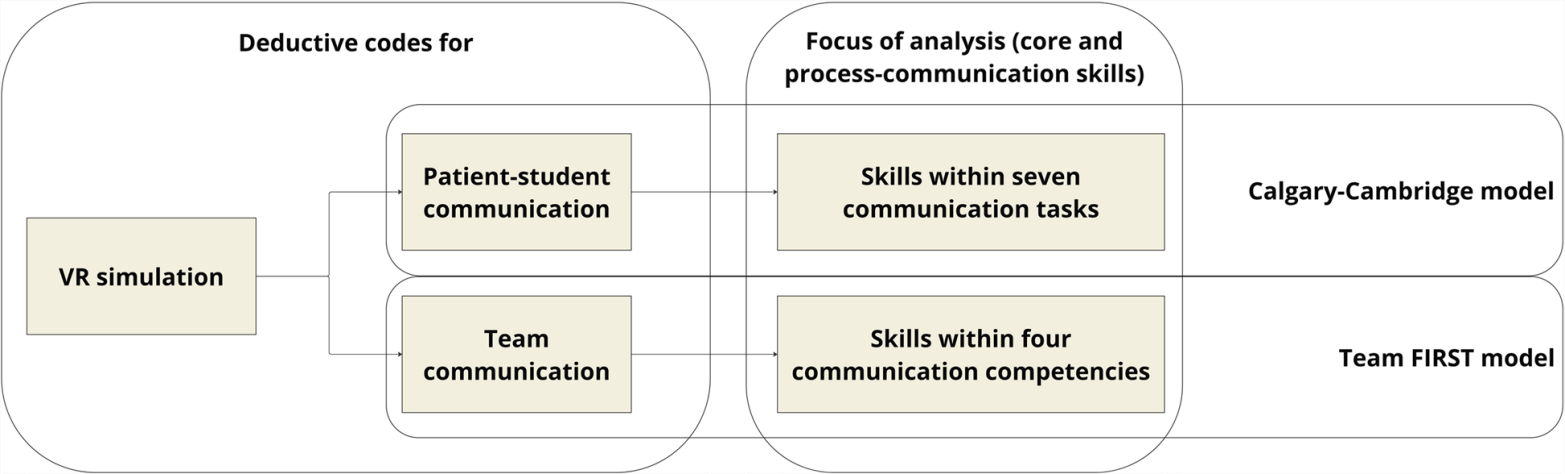
Deductive coding process for communication skills in VR simulations, following communication models (i.e., Calgary-Cambridge model [1], Team FIRST model [29])

For the deductive coding approach, we developed a coding manual (Appendix 2) based on two communication models [1, 29]. We chose the Calgary-Cambridge model for its comprehensive skills set within patient-student communication, framed within the seven communication tasks of a patient-physician consultation (i.e., initiating a session, gathering information, physical examination, sharing information and planning, closing the session, building a relationship, providing structure). Though semi-acute, the scenario required students to perform these communication tasks. We chose the Team FIRST model for its clear, separate communication competencies for team communication in healthcare (i.e., closing the loop in communication, structuring communication, sharing unique information, asking clarifying questions). We created codes for core communication skills (i.e., which micro skills are used, such as paraphrasing and asking questions) and process communication skills (i.e., how these micro skills are used, for example, to inform or reassure) within patient-student and dyadic team communication (Fig. 4). While exploring transcripts, we generated codes for noteworthy communication behaviors (e.g., interrupting someone). While coding, we co-coded skills/behaviors with their surrounding interactions to capture the communication content and context.

Second, to ensure triangulation, two researchers independently coded 10% of transcripts based on the coding manual. Interrater reliability showed disagreements for 3 of 47 simulation codes. Raters discussed codes until they reached consensus. The other 90% of transcripts were subsequently coded by the primary researcher. Third, we analyzed skills and behavioral codes and explored overarching communication patterns in VR simulation. Fourth, we developed themes for VR-mediated CST. Patterns and themes were discussed in a workshop with three researchers and two simulation teachers. Lastly, themes were refined, defined, and grouped in the multidisciplinary study team.

**Fig. 5:**
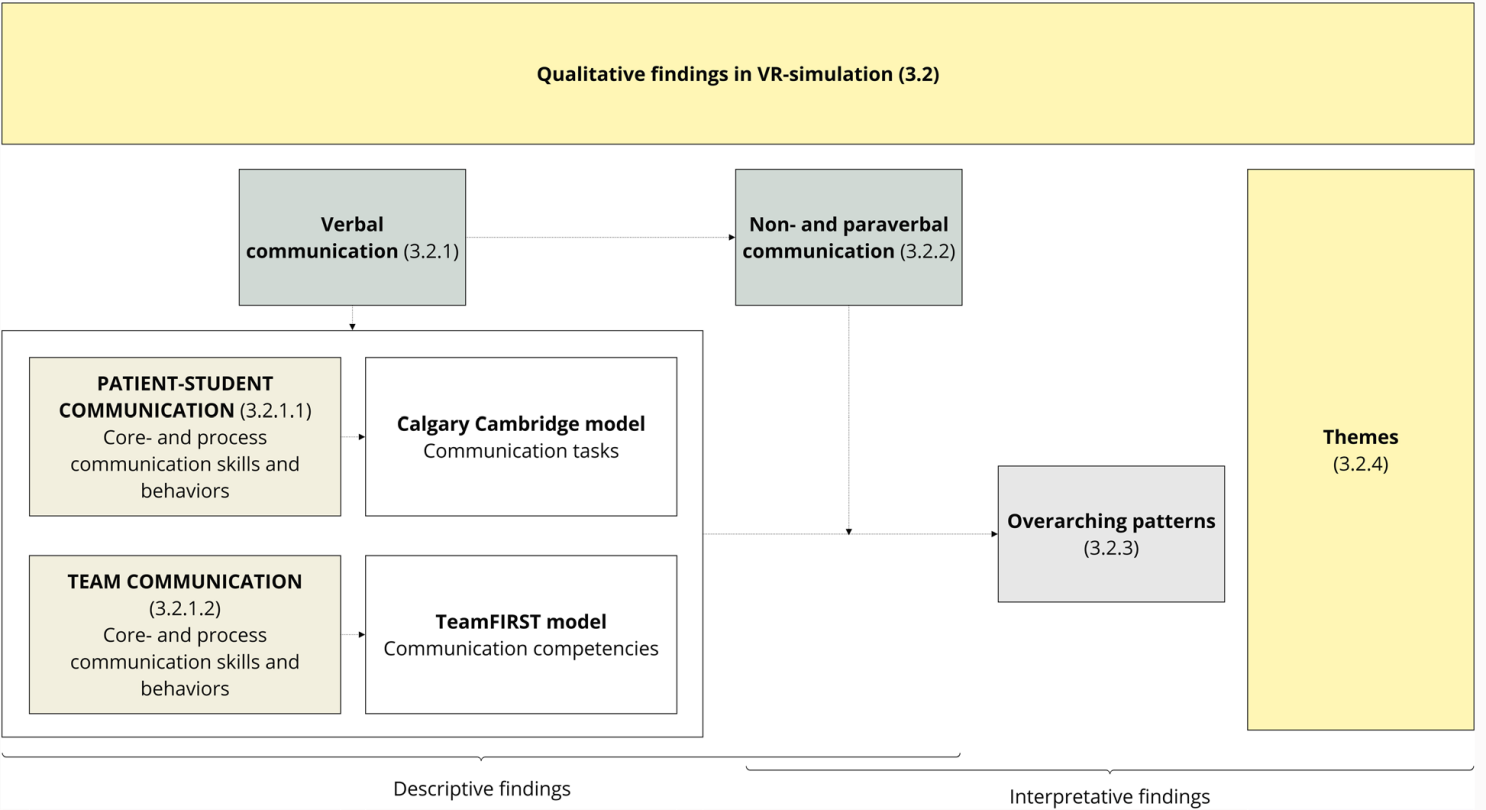
Structured approach to the presentation of descriptive and interpretative findings for patient-student and team communication in a dyadic VR simulation

We structured our findings (1) by describing verbal skills and noteworthy content within the Calgary-Cambridge and Team FIRST model (Fig. 5). Then, we described nonverbal and paraverbal behaviors (2). Next, we described (3) overarching communication patterns. Finally, we described (4) themes for dyadic VR SB-CST.

## Results

### Demographics

A group of 13 female and 9 male students between the ages of 19 and 25 participated in this study. Twelve students had experience with at least 1 VR game; 10 students had no VR experience. TF, a clinical teacher and nurse, had 5 years of experience guiding simulations. The summary of demographics is shown in Appendix 3.

### Qualitative findings

#### Verbal communication

##### Patient-student communication

**Fig. 6:**
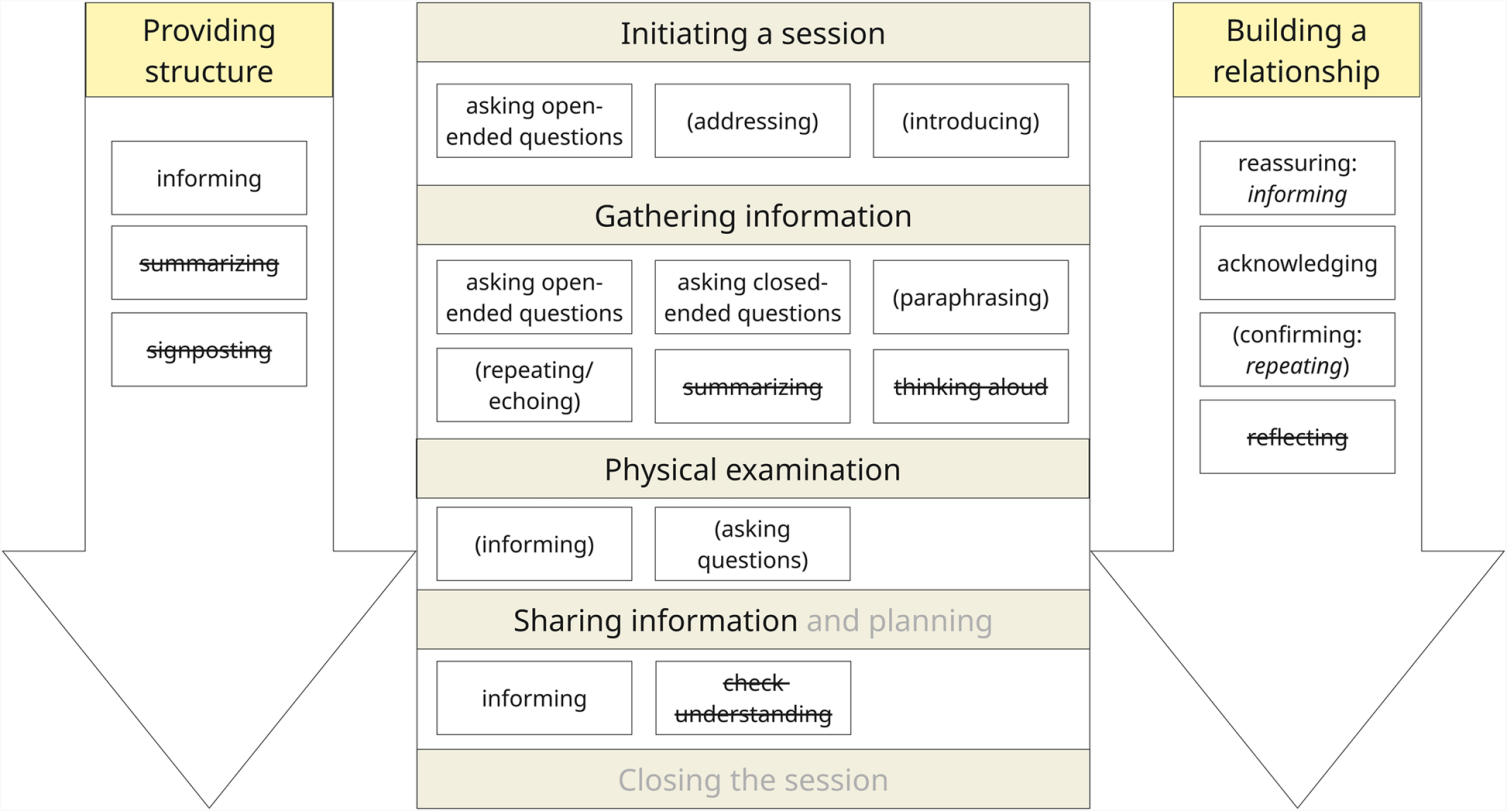
Observed core and process communication skills within the seven communication tasks of the Calgary-Cambridge model. Crossed out skills were expected within the model, but not observed in VR simulation. Bracketed skills were observed sometimes. Other skills were regularly observed. Planning and closing the session were absent (gray) tasks due to the semi-acute scenario and the study design

We found that when analyzing patient-student communication, skills to open communication (e.g., paraphrasing, reflecting), to structure communication, and to build the relationship were often inadequate or absent. We found that students focused on the task of gathering information. Students preferred informing and asking questions within the communication tasks (Fig. 6).

Students initiated the simulation variably. Most pairs asked an open-ended starting question; however, they rarely addressed or introduced the patient and almost immediately continued to team communication. A correct initiation was rare, with some pairs skipping this task entirely.

To gather information, students mostly asked questions. A gradual progression from open-ended to closed-ended questions during the orientation phase was predominantly absent. We observed some open-ended questions covering large-response areas, mostly limited to the orientation phase. Students quickly switched to closed-ended questions covering small-response areas (e.g., “when,” “where,” “which” questions). Closed-ended questions mostly explored the patient’s complaint from a biomedical perspective. Questions exploring the patient’s perspective were limited, mostly closed-ended, and triggered by the patient. We observed some students paraphrasing and repeating to clarify or to encourage, but not as preferred skills. Some students asked suggestive questions. Thinking aloud or summarizations were absent.

Students rarely asked permission for physical contact. Some students informed the patient about physical contact, but without asking for permission.

Students shared information mostly after patients’ questions or attention-seeking behaviors (e.g., vocalizing distress). Students mostly informed task-orientedly in one or two sentences, continuing physical examination or team communication. Students rarely informed about probable causes and only after patient initiation. Some students did not explain terminology to the patient (e.g., “electrocardiogram,” “parameters”). Informing was also used as a preferred reassurance strategy (e.g., “not to worry”) in relation-oriented communication. Students did not check the patients’ understanding.

To provide structure, students did not summarize or signpost. Instead, they offered short-term insights in the consultation by informing, almost exclusively, patient-initiated.

To build a relationship, students rarely reacted to patient’s expressed emotions or concerns. When they did, reactions took the form of informing (intended to reassure), acknowledging, or confirming (e.g., repeating patient). Reflecting was absent.

##### Team communication

**Fig. 7:**
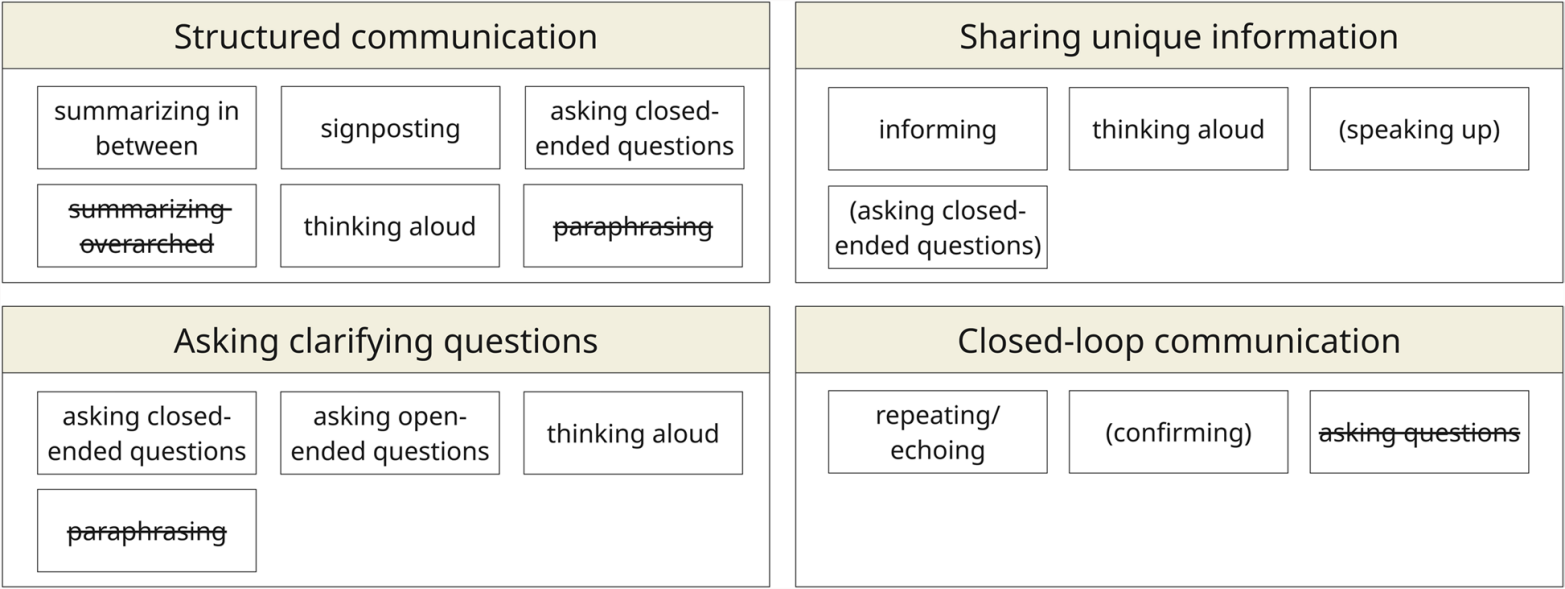
Core and process communication skills within the four communication competencies of the Team FIRST model. Crossed out skills were expected within the model, but not observed. Bracketed skills were observed sometimes. Other skills were regularly observed

For team communication, we found that skills to open communication (e.g., speaking up, paraphrasing) and to close the loop in communication were insufficient or absent. Analysis showed a focus on the competencies of structuring and information gathering/sharing. Students preferred thinking aloud and asking questions within communication competencies (Fig. 7).

Students structured communication with regular intermediate summaries (e.g., within ABCDE elements [airway, breathing, circulation, disability, exposure]), rarely with overarching summaries. All pairs were signposted in order to structure the examination (e.g., ABCDE approach). This was regularly accompanied by closed-ended questions (e.g., confirming), thinking aloud (e.g., task division), or intermediate summaries. Paraphrasing was absent.

Clarifying questions (e.g., for tasks, knowledge, opinions/doubts) were closed- and open-ended questions with small (e.g., “what,” “when”) and large response areas (e.g., thinking aloud). We prominently observed open- and closed-ended questions for communication about technological aspects. Again, paraphrasing was also absent.

Students were informed to share unique information (e.g., about parameters, nudges, tasks). Thinking aloud was observed regularly, sometimes accompanied by speaking up or closed-ended questions about doubts (e.g., “I think,” “in my opinion”). Students also regularly informed their peers about technological content.

If students closed the loop, they did so by repeating/echoing or confirming, but students often continued immediately without waiting for confirmation. Students did not ask questions to close the loop.

#### Nonverbal and paraverbal communication

We found that the use of nonverbal and paraverbal skills/behaviors was mostly unintended.

Conversational *silences* were not observed as intentional nonverbal skills. Silences of different durations (e.g., 2–5 s, 5–10 s, and > 10 s) regularly arose in team communication during technical issues and clinical reasoning. We did not identify intentional use of these silences as a conversational tool for patient-student communication. Silences were not used meaningfully and were eventually replaced by team communication.

We observed *intonation* parameters (paraverbal behavior) but with unclear intent. Some students changed volume, speaking louder with patients and softer with peers. Some students changed their clarity of speech, being clearer with patients and mumbling with peers. Some students used flat intonation when verbally acknowledging patients, creating a discrepancy between content and intention; therefore not conveying authentic, empathic relation-oriented communication.

#### Overarching communication patterns

Analysis revealed five overarching patterns for communication in VR simulation.

First, we found that VR-related technical and nontechnical issues influenced the *orientation of communication*, more specifically in the form of informal student communication (e.g., laughing), teacher/researcher communication (e.g., asking for support), and unclear orientation (e.g., no clear receiver).

E22: So, we're going to start with A.

N12: Wait. I'm not there yet.

E22: Oh, you're at the door. (*laughing*).

Within the orientations, we identified *prominent team communication* in all student pairs. Patient-student communication characterized most scenario starts, decreasing to shorter intervals throughout simulations. At scenario endings, if no cause was found, patient-student communication often increased (e.g., asking questions).

We also identified *limited relation-oriented communication* within patient–student and team communication. In patient-student communication, this was marked by behaviors such as not picking up on patient cues and responding generically to patients’ emotions or concerns. Students mostly ignored the patient, sometimes responding to emotions or concerns by informing, confirming, and asking questions.

Patient: °It's my lungs, huh?°

S22: (*talking to fellow student, ignoring patient*) For me it says (*reading nudge*) heart is normal.

V12: Yes, for me… °I actually don't see anything ()°.

In team communication, this pattern was only observed in skills such as speaking up about doubt and asking for opinions. Reflecting or acknowledging emotions was absent.

H26: [Maybe] can we, uhm.

M16: Put in an IV?

H26: Take blood or something?

M16: °I don't really know°

Subsequently, we identified *verbal communication overload*, observed in students engaging verbally in multiple orientations of communication, and different contents, such as simulation tasks and technological issues. Skills to open communication (e.g., reflecting, speaking up) were less present. Instead, skills often closed communication (e.g., closed-ended questions), therefore eliciting more verbal communication.

Lastly, we identified a noteworthy pattern of *altered conversational turn-taking* with unclear intent of nonverbal and paraverbal communication and in behaviors such as speaking simultaneously (indicated by “[]”), where students regularly interrupted a conversation partner or spoke in parallel.

Patient: [Ooh, what do you think it is]

L17: [But, then you can see that in the blood.] We're trying to find out, sir, but [() you're in pain.]

A27: [We're not sure yet] We're going to find out, sir.

#### Themes for VR SB-CST

Analyzing these patterns, behaviors, and skills, we generated two themes concerning CST in VR simulation.

##### Focus of attention for CST

Students focused their attention on communication skills related to team and task-oriented communication, with a primary emphasis on verbal skills. In contrast, patient-student and relation-oriented communication received less attention. Additionally, we observed multiple orientations of communication, including people talking simultaneously or failing to respond to one another, interruptions, technological issues, and altered auditory-visual experiences (e.g., patient-avatar), as well as verbal communication overload and, therefore, increasing students’ cognitive load. Collectively, these VR- and non-VR-related elements impacted students to (un)consciously shift their focus of attention toward certain communication orientations and skills.

##### Flow of CST

Communication flow was observed in both team and patient-student encounters (e.g., both students simultaneously engaging in patient communication). However, regular disruptions (e.g., silences, interruptions, unclear intentionality of para/nonverbal skills, altered conversational turn-taking) were also present. They were connected to delays in information delivery (e.g., written nudges), technological issues, unfamiliarity with VR, and cognitive demands of clinical reasoning. Additionally, multiple orientations disrupted the primary patient-student and team communication. Collectively, these VR- and non-VR-related elements impacted the flow of CST.

## Discussion

Our study investigated medical undergraduates’ communication skills in a dyadic VR simulation, revealing how VR alters the focus of attention and flow of CST in simulation. The impact is reflected in three major findings.

A first key finding was that students prioritized team over patient-student communication during multiuser VR simulations. Within both team and patient communication, students favored communication on tasks over communication supporting the interpersonal or professional relationship (e.g., speaking about concerns, emotions). To communicate in medical encounters, students preferred asking questions and informing. The VR context, however, shaped the prioritizations in communication. Firstly, the characteristics of the avatars mattered. Dynamic (student) avatars, with facial expressions and body movement, encouraged communication. Fixed (patient) avatars, lacking nonverbal signals, reduced connectedness (e.g., difficulty detecting patient signals) and, therefore, might have impeded communication [30]. The altered perception of nonverbal behavior might have limited students’ engagement to interpret patient signals, reducing the authenticity of the interaction and, therefore, altering patient-student communication. Even though the virtual patient remained communicative (auditory), visual authenticity seemed to be instrumental. Secondly, it appeared that the scenario also mattered. In semi-acute bedside settings, the urgency of a complaint and the team setting might have prompted students to prioritize task communication (e.g., gathering information) and to engage patients less [31]. Additionally, team and task communication might have felt safer than dealing with a patient’s emotions or concerns [32]. Overall, the VR context altered the focus of attention toward team and task communication.

A second key finding was that conversational turn-taking changed in multiuser VR simulation. Students often spoke simultaneously, in parallel, or interrupted each other. Technology influenced how conversational cues were perceived and thereby impacted communication behaviors. Visual nonverbal behaviors, such as eye contact, were presented virtually, which may have impacted signal detection and interpretation for turn-taking. Even though VR used a real-world speaking voice (i.e., not through headphones), auditory nonverbal skills, such as silences, were used unintentionally, which additionally influenced conversational turn-taking. Therefore, interaction in VR appeared more focused on verbal communication [18]. Overall, VR technology influenced observation and interpretation of conversational cues and, subsequently, communication flow.

A third key finding was that VR impacted students’ cognitive load. VR added or altered experiential and technological elements to simulations. Moreover, first-time VR might have impacted experienced load. This could possibly have caused students to prioritize task and team communication. Additionally, there was a verbal communication overload, observed in students engaging verbally in different contents, with communication skills that evoked closed communication. Although the exact influence of VR was unclear, this heightened verbal load was instrumental. Subsequently, cognitive overload created by VR could have distracted focus and flow of communication and impacted learning outcomes [33].

While VR simulations seem to show potential for primarily team and task communication training [34], their potential for patient, interpersonal, or interprofessional communication is unclear. We propose three recommendations on the contextual design of VR SB-CST in order to optimize conditions for CST for enhanced focus of attention and flow. First, more emphasis should be placed on the avatar design. An avatar with dynamic nonverbal behaviors, such as facial expressions and eye contact, can stimulate observation and learner engagement. In patient-student encounters, this can guide communication focus to the patient. Second, the scenario should relate strongly to the communication training goals to focus attention. Semi-acute settings might demand different communication priorities compared to consultation settings. If the acquisition of patient-student communication is the training goal, perhaps semi-acute bedside settings are not the primary scenarios of choice. Third, the instructions for VR simulation need to be framed clearly to control for cognitive load. A screen-based simulation observation by the TF can impact teacher/researcher communication and support during the simulation. TF might miss nonverbal behaviors, therefore limiting guidance. Pretraining of students in the VR environment is, therefore, important. Additionally, guidance toward expectations of paraverbal and nonverbal communication is key. However, repeated practice for familiarization is instrumental. Carefully designed instructions and pretraining might decrease distraction and increase the likelihood of flow in communication [24].

## Strengths and limitations

Our studies’ strength lies in the aim to analyze specific communication skills in VR simulation. To our knowledge, this is the first study exploring core and process-communication skills in a dyadic, HMD-VR simulation. Our deductive approach enabled us to guide the analysis of these communication skills. Our inductive approach enabled us to reveal additional unexpected communication behaviors and patterns. We used established communication models to interpret our observations. Furthermore, we investigated core communication skills in team communication, where most team communication models do not include core skills.

The study has some limitations impacting generalization of our findings. Limited nonverbal and paraverbal communication skills were collected and analyzed, constraining a more holistic interpretation of communication. Only one scenario was performed, possibly determining the presence/absence of communication skills by urgency and patient characteristics. The TF was a familiar clinical teacher to the students, creating possible bias toward interaction. Students were all facilitated by the same teacher; however, the design with only one TF might bias the generalizability of the results. Students entered the study voluntarily, which may indicate a highly motivated or communicatively strong sample or a sample with a particular interest in VR and/or simulation. The primary researcher is a communication trainer, both familiar with teaching and assessing students’ communication skills. Students were never trained and assessed by the primary researcher, minimizing bias. To further minimize bias, data were analyzed through an observational lens. Additionally, regular steps were taken back to reflect.

## Implications for future research

This study offers a first, exploratory step in communication-focused multiuser medical SBE. A next step is to investigate communication content and skills within the debriefings of VR simulations. To further expand our insights in VR, research should compare the impact of VR on communication skills in dyadic VR simulations and non-VR simulations, to create an understanding of differences and reasons for impact. Another recommendation is to analyze prebriefing data and non/paraverbal skills (e.g., with objective measurements of eye contact, facial expressions, intonation), to gain a holistic understanding of patient-student and team communication in VR.

## Conclusion

Our findings contribute to a deeper understanding of how multiuser VR impacts SB-CST among medical undergraduates. While VR offers opportunities for experiential learning, it also presents limitations for SB-CST. Dyadic VR simulations lead to the prioritization of intraprofessional communication over interpersonal communication. VR increases students’ cognitive load, potentially diverting their focus of attention to and disrupting the flow of CST. While the specific impact of VR cannot be conclusively determined, altered paraverbal and nonverbal communication and altered conversational turn-taking cannot be overlooked. Given the critical role of paraverbal and nonverbal communication for interpersonal communication, it is unclear whether VR-SBE is suitable for empathic CST. The contextual design of avatars, scenarios, and instructions is instrumental to the future use of VR for CST. Additionally, further investigation into the role of debriefing in VR SB-CST is necessary.

## Supplementary Information

Supplementary Material 1: Appendix 1. Transcription notations.

Supplementary Material 2: Appendix 2. Coding manual – *VR simulations.*

Supplementary Material 3: Appendix 3. Demographics of participants, collected through qualitative surveys.

## Data Availability

The dataset(s) supporting the conclusions of this article are available from the corresponding author upon motivated request, accompanied by a statement of purpose.

## Abbreviations

CST: Communication skills training
HMD: Head-mounted display
SB-CST: Simulation-Based Communication Skills Training
SBE: Simulation-based education
SCOT: Smart Collaboration Tutor
TF: Teacher-facilitator
VR: Virtual reality
