# Supplementary material for: Training communication skills in a multiuser medical virtual reality simulation: a qualitative, observational study
Source: Adv Simul (Lond). 2025 Nov 24;10:59. doi: 10.1186/s41077-025-00386-8 (PMC12642034; doi:10.1186/s41077-025-00386-8)
Supplement: Supplementary file 1 — Supplementary Material 1: Appendix 1. Transcription notations. [file 41077_2025_386_MOESM1_ESM.docx]

**Appendix 1**

A selection of transcription notations for simulation transcripts were used to indicate observations in sequencing, intervals and prosodic features. These are adopted from this reference:

White SJ. Conversation Analysis: An Introduction to Methodology, Data Collection, and Analysis. In: Liamputtong P, editors. Handbook of Research Methods in Health Social Sciences. Singapore: Springer; 2019:471-490.

On their turn, these notations are based on the Jeffersonian transcription system, and taken from:

Gardner R. Conversation analysis. In: Davies A, Elder C, editors. The handbook of applied

linguistics. Oxford: Blackwell; 2004. p. 262–84.

ten Have P. Doing conversation analysis. London: Sage; 1999.

| Transcription notations | Description |
| --- | --- |
| Transcriber’s doubts notations | |
| ( ) | *Parentheses are used when a part of the conversation was unclear to the transcriber.* |
| SEQUENCING notations | |
| [ | *A left bracket is used to indicate the start of observable overlap in conversation between two or more speakers.* |
| ] | *A right bracket is used to indicate the end of observable overlap in conversation between two or more speakers.* |
| = | *Equal signs are used when there is no gap in turn-taking. One equal sign is placed at the end of one line and one at the beginning of the next line. (latching)* |
| INTERVAL notations | |
| (0.0) | *Numbers in parentheses are used to indicate the duration of silences in between utterances of the same or different speakers. In the coding process durations are grouped.* |
| PROSODIC FEATURES notations | |
| - | *A dash indicates a cutoff in a conversation when one stops talking. (interruption)* |
| °word° | *To indicate more quiet utterances or parts of a conversation, degree signs are used to bracket the more quiet parts.* |
| WORD | *To indicate more loud utterances or parts of a conversation, capital letters are used.* |
| <word> | *To indicate slower speech, left/right carats are used to bracket an utterance or part of a conversation.* |
| >word< | *To indicate faster speech, right/left carats are used to bracket an utterance or part of a conversation.* |
