# Supplementary material for: Training communication skills in a multiuser medical virtual reality simulation: a qualitative, observational study
Source: Adv Simul (Lond). 2025 Nov 24;10:59. doi: 10.1186/s41077-025-00386-8 (PMC12642034; doi:10.1186/s41077-025-00386-8)
Supplement: Supplementary file 2 — Supplementary Material 2: Appendix 2. Coding manual – VR simulations. [file 41077_2025_386_MOESM2_ESM.docx]

**Appendix 2**

**CODING MANUAL –** *VR-SIMULATIONS*

*When in doubt of giving a certain code, an annotation will be added with the intended code, but the utterance will not be coded itself.*

*CODES for RELATIONAL ORIENTATION OF COMMUNICATION*

*With the following codes we observe relational orientations of communicative interactions.*

| **RELATIONAL ORIENTATION OF COMMUNICATION** | |
| --- | --- |
| **Code 1: Professional patient-student communication** (student in physician role) | |
| Code 1**a** | |
| Label | **Patient-initiated patient-oriented communication** |
| Definition | Patient-student communication (**bloc**), initiated by the patient. Initiation is identified as a question or utterance by the patient. The utterance must be more than a vocalization of the patient (f.e. ‘oooh’ or ‘pff’). The following response by the student is required. |
| Description/**excerpt** | P: Is there really nothing that can be done to make the pain start to get better?  A27: °Well, we're going to try our best, right°. And where, do you still have [pain somewhere in your chest, on your shoulders and such?] |
| Code 1**b** | |
| Label | **Student-initiated patient-oriented communication** |
| Definition | Patient-student communication (**bloc**), initiated by the student. Initiation is identified as a question or other utterance by the student AND a following response by the patient (f.e. checking in with patient how he is doing after medication, asking anamnestic questions, speaking aloud that blood will be taken) |
| Description/**excerpt** | H25: Can we take a look at your back, sir?  P: Yes, yes, but be careful, I really can't tolerate much, not much here.  M15: And that shortness of breath, is that mainly when sitting or standing [or lying down?]  P: [Yes, it's almost] continuous.  M15: Almost continuous in all positions actually.  P: Yes, really, a tight feeling, that pressure, really, ooh.  M15: That pressure, yes. |
| **Code 2**: **Professional team communication** | |
| Label | **Team oriented communication** |
| Definition | Collaborative communication (**bloc**). Communication aimed at or with fellow students. Orientation is identified when addressing the fellow student, using the students’ name, asking a medical question, an utterance regarding clinical reasoning… (f.e. summing up the gathered information, speaking aloud about what they are doing) |
| Description/**excerpt** | B19: °A little, like, dyspnea and that shortness of breath, a bit like heart failure, huh.°  E29: Yes.  P: Oh, but really that pressure on my chest is hard. That doesn't improve.  E29: It's strange that it doesn't go away then.  B19: Yes, and the rhythm is also normal.  E29: °Uhm, that tachycardia remains a bit.°  B19. Yes. That weak pulse may also be a bit underfilled, but we're not allowed to give fluids if it's heart failure [that's quite dangerous]  E29: [Yes.] But the blood pressure has dropped a bit now.  B19: Dropped.  E29: 118 over 75  B19: Yes. (2.5) |
| **Code 3: Teacher/researcher communication** | |
| Code 3**a** | |
| Label | **Student-initiated teacher-oriented communication** |
| Definition | Communication aimed at the teacher or researcher present during simulation activity (‘speaking to god’) (**bloc or part**). Orientation is identified when asking a question to the teacher/researcher, clear because of name, content, register (paraverbal), initiated by the student. |
| Description/**excerpt** | I21: There was a text there just now, but I couldn't read it, and when I could start reading it, it just disappeared.  Teacher: It was that you ( ) purple boxes all the info ( ) you need-  L11: By pressing it  I21: Ah, okay, like that. |
| Code 3**b** | |
| Label | **Teacher-initiated teacher-oriented communication** |
| Definition | Communication aimed at the student during simulation activity (‘speaking to god’) (**bloc or part**). Orientation is identified when the teacher/researcher speaks form this function to the student. It is clear because of name, content, register (paraverbal. |
| Description/**excerpt** | Teacher: That's an ECG electrode. (reading text nudge) (2.5)  M24: Ah, here's the saturation.  R14: (laughing) |
| **Code 4:** **Informal student communication** | |
| Code 4a | |
| Label | **Informal student-oriented communication (related to tech issue or error)** |
| Definition | Communication clearly aimed at a fellow student as a friend, not as a team member (**bloc or part**). Orientation is identified because of content, different word choice (informal), changing non-verbal/paraverbal features, laughing. |
| Description/**excerpt** | L11: The saturation is 91. That's not that high. (2.5) Okay, so airway is normally fine, because the gentleman is talking, right? Oops, oops, I'm so close  I21: (laughing)  L11: °I'm going to go stand on the other side° |
| Code 4b | |
| Label | **Informal student-oriented communication (not related to tech issue or error)** |
| Definition | Communication clearly aimed at a fellow student as a friend, not as a team member (**bloc or part**). Orientation is identified because of content, different word choice (informal), changing non-verbal/paraverbal features, laughing. |
| Description/excerpt | P: Ooh, what's wrong with me.  N12: We're still investigating, Danny.  E22/N12: (laughing)  N12: Now we're not finding anything.  E22: I really don't know.  P: Ooh |
| **Code 5** | |
| Label | **Unclear or no orientation (by student)** |
| Definition | Unclear orientation of communication (**bloc or part**). Orientation (code) is identified when content isn’t clearly pointing out a receiver and a receiver is not clearly addressed or no clear changes in paraverbal features are present (used register); or when the student seems to be speaking to one’s self. Additional notes of observer can be found between brackets and in smaller text to give more context. |
| Description/**excerpt** | P: Ooh. (3.0)  L11: Breathing. Can I listen to the lungs like that, or? Look at the breathing°frequency° (10.5) Should I just press those purple boxes?  Teacher: Yes |

*CODES for COMMUNICATION SKILLS*

*The following codes are for core and process-communication skills in team and patient-student communication [1, 24].*

| **VERBAL SKILLS by students** | |
| --- | --- |
| Code 6 | |
| Label | **Paraphrasing** |
| Definition | Rephrasing in one’s own words the content of what the other person said. Paraphrases can follow an utterance immediately or postponed. |
| Description/**excerpt** | P: Well, yes, just now at work when I was shoveling concrete, it started and it won't go away now.  V21: No, just because you're resting now doesn't mean it's any less bad. It's still= |
| Code 7 | |
| Label | **Repeating or echoing** |
| Definition | Repeating or echoing some words or long phrase. Repetitions/echos can follow an utterance immediately or postponed. |
| Description/**excerpt** | E22: Heart rate normal, a little soft.  N12: °Heart rate normal, a little soft.° |
| Code 8 | |
| Label | **Open questioning** |
| Definition | Asking an open ended question, where a longer answer is expected to follow. These are questions starting with an interrogative (f.e. what, where, who, when…) or questions inviting a person to elaborate on something (f.e. Could you tell more about that?). |
| Description/**excerpt** | (small response area)  A11: [Then], the saturation, what is it like at the moment?=  V21: =92.  OR  (large response area)  E28: Ok, [blood taken] What does our lab actually say?  P: [( )]  A18: What can we do then? (laughing) (4.0)  E28: So, those different troponins.  A18: Yes.  E28: Lab is not yet known (nudge) |
| Code 8a | |
| Label | **Bloc of sequential open questions** |
| Definition | A sequence of two or more open questions (picking up on what the patient says), with following answers of the patient to these questions. |
| Description/**excerpt** | V21: We are now measuring all your parameters. How are you feeling, sir?  P: Ooh. I am so short of breath.  V21: Short of breath. And when did that start anyway? |
| Code 9 | |
| Label | **Closed questioning** |
| Definition | Asking a closed ended question, where a short answer or yes/no-answer is expected to follow. These questions start with a verb (f.e. Is is painfull?). |
| Description/**excerpt** | V21: And have you had any health problems before? You've said before that you've had shortness of breath before.  P: Yes, I have, I've had that now and then in the past few months, that I had that pain in my chest (…), but that went away when I rested, when I stopped. |
| Code 9a | |
| Label | **Bloc of sequential closed questions** |
| Definition | A sequence of two or more closed questions (sometimes perceived as a *question fire*), with following answers of the patient to these questions. |
| Description/**excerpt** | A11: And does that radiate to somewhere?  P: Oh yeah, yeah a little bit, yeah, yeah.  A11: And to where exactly?  P: Uhm, to, to the left. (1.5) |
| Code 10 | |
| Label | **Summarizing** |
| Definition | Recapitulating the essential elements of what has been said before (larger timeframe than paraphrasing) in one’s own words. This can be done by one students or two students together. |
| Description/**excerpt** | O16: A little ( ) Hmm. Okay, so we have blood pressure, ECG, heart rate [and capillary refill was also normal.]  H26: [Capillary refill is normal] We might be able to take an ECG sometime, because he does have chest pain. |
| Code 11 | |
| Label | **Reflecting** |
| Definition | Rephrasing in one’s own words the observed emotions of the other person whom the utterance is aimed at. Inherently this is a bit interpretative, but the interpretation must be suited to the emotions observed. This reflection is not formulated as a question and can follow an utterance immediately or postponed. |
| Description/**example** | Patient: Can't you really tell me what's wrong? I'm running out of patience.  Student: You feel frustrated that you don't know anything. |
| Code 12 | |
| Label | **Informing** |
| Definition | Giving information about something or what is about to happen a patient (f.e. about what is going to happen) or student (f.e. text information only one student sees in the VR-environment; a nudge is given). |
| Description/**excerpt** | E29: Yes, we'll ask about it later, but we want to do a thorough investigation first, so that we know for sure that there's nothing wrong with you.  P: Ooh. Something's wrong, huh, I feel that something's not right. |
| Code 13 | |
| Label | **Verbally acknowledging a patient** |
| Definition | Verbal acknowledging of a patients thoughts or feelings by statement or short utterance. There is no difference made in authentic or non-authentic in this code.  Caution: Authenticity is not withdrawn in this code, but will be researched in the next step. |
| Description/**excerpt** | R14: Yes. [Yes. But look], we will do everything to see if we can rule it out or not. Okay. °Look, in the meantime my colleague is going to stick a few more electrodes on you.°  Patient: Yes, yes. it's so uncomfortable.  R14: Hmm. I get it, hmm. And, do you have, do you have family that can be contacted, or? |
| Code 14 | |
| Label | **Signposting** |
| Definition | Speaking aloud to move on to the next step and/or what this next step is, to give structure to a conversation. This can be f.e. continuing in a step of ABCDE or continuing to another examination. |
| Description/**excerpt** | A18: =So airway is okay.  E28: Yeah.  A18: Uhm. Breathing then. (3.5) Uhm, wait, it's all gone for a while °( )°. Uhm, three things were in there. |
| Code 15 | |
| Label | **Thinking aloud** |
| Definition | Saying out loud what one is thinking at that moment, regarding opinions, questions, reasoning. It is not the same as informing where information is shared about a procedure or nudge. **(part or bloc)** |
| Description/**excerpt** | E29: Yeah, °I don't know either.°  B19: °It's not really something very threatening°  E29: Yeah, but. Indeed, but that tachycardia remains. [( )] to do something about it  B19: [What would you-] Hmm. What would you give for that, if it were increased?  E29: Uhm (4.0)  B19: Maybe the tachycardia is a bit of compensation for the heart failure. |
| Code 16 | |
| Label | **Speaking up** |
| Definition | Speaking up to a fellow student when something is unclear or when something seems wrong or when doubt about something is mentioned aloud. |
| Description/**excerpt** | V12: Yeah, [97.]  S22: [Okay] Let's move on, circulation.  V12: ( ) His blood pressure is 11 °over 7.°  S22: °Okay, that's not really normal°  V12: That is, yes, it was a lot higher just now, so, °so it's gone down a lot.° |
| Code 17 | |
| Label | **Giving an order to a fellow student** |
| Definition | One student gives a task or order to the other student; not by asking, but as an announcement. |
| Description/**excerpt** | O26: [Uhm], are you going to, or should I maybe °measure° the saturation? If that's okay?  M16: (Uhm] Yeah, ah there. Wait hey.= |
| Code 18 | |
| Label | **Addressing/introducing a person** |
| Definition | Addressing/introducting the patient or a fellow student **with name, prefix or function**. |
| Description/**excerpt** | E22: Hi Danny, how are you? Tell me, what happened?  P: Yeah, how are you. Not good, huh? Oh, I really don't feel good. |
| **NON-VERBAL SKILLS by students** | |
| Code 19 | |
| Label | **Silence of 2 to 5 seconds** |
| Definition | The silence takes 2 to 5 seconds (5 not included). This could be interpreted as the standard rule, but interpretation of this ‘purposeful’ silence needs to be checked in the data. It may also be just a silence of 3-5 seconds. |
| Description/**excerpt** | L11: Yes, for the pain, huh=  I21: =against the pain, erm (2.5)  L11: Shall we give an injection? (2.5)  I21: Injection, erm, can't we give it first, rather something like medication, Dafalgan or something, first? |
| Code 20 |  |
| Label | **Silence of 5 to 10 seconds** |
| Definition | The silence takes 5 to 10 seconds (10 not included). |
| Description/**excerpt** | A11: =I'm going to take a good look at the ECG to see if we can see °certainly nothing there°. And can we do a 12-lead ECG or is that not possible? (6.5)  V21: Heart rate is 105. |
| Code 21 |  |
| Label | **Silence of more than 10 seconds** |
| Definition | The silence takes more than 10 seconds (f.e. while waiting on a nudge). |
| Description/**excerpt** | L11: And so it's really in your chest that you have pain.  P: Yeah, yeah, I'm also short of breath. Ooh.  (11.0)  P: Ooh, what are you doing now? |
| **PARAVERBAL SKILLS by students (observations)** | |
| Code 22 | |
| Label | **Speaking in soft(er) volume** |
| Definition | A student speaks remarkably soft or softer with fellow student or patient, not because of the student turning away from the recording camera (and so microphone). |
| Description/**excerpt** | P: I have chest pain, huh. Ooh.  A18: °( )° (5.0) Uhm, yeah, °that actually looks pretty normal to me too. Can you do multiple leads? No.°  E28: °Maybe like this.°  A18: °No.°  E28: °Oops, I'm getting a text, I can't see it properly.° |
| Code 23 | |
| Label | **Speaking in loud(er) volume** |
| Definition | A student speaks remarkably loud or louder with fellow student or patient, not because of the student turning towards the recording camera (and so microphone). |
| Description/**excerpt** | R13: And what do you mean, really not good?  P: Ooh, my chest hurts so much and I'm so uncomfortable.  R13: CHEST PAIN.  P: Yes.  R13: AND WHAT KIND OF PAIN? |
| Code 24 | |
| Label | **Speaking fast(er)** |
| Definition | A student speaks remarkably fast or faster with fellow student or patient. |
| Description/**excerpt** | P: Yeah, a little short of breath. And yeah, I'm so uncomfortable. Ooh, it's not going well.  M23: >It's not going well<, okay.= |
| Code 25 | |
| Label | **Speaking slow(er)** |
| Definition | A student speaks remarkably slow or slower with fellow student or patient. |
| Description/**excerpt** | P: Ooh. How long is this pain in my chest going to last? It's so annoying. (5.5)  R13: We're going to help you as soon as possible. We're going to ( ) look at <your heart rate and, heart, uhm>, ECG. (3.0)  P: Ooh |

*Notes:*

*Humming is not included. Smiles are not included in non-verbal listening skills, because it is impossible to register as an observer (i.e. VR-glasses). Touch is not included in non-verbal listening skills, because it is impossible to register as an observer (i.e. controllers). Eye contact, specifically, is not included in non-verbal listening skills, because it is impossible to register as an observer (i.e. VR glasses). Mimics/facial expression is not included in non-verbal listening skills, because it is impossible to register as an observer (i.e. VR-glasses). Open posture is not included separately, but annotations can be made.*

*Nodding, forward leaning and visual attention, supporting hand gestures: not able to be consistent in video observations… 🡪 Annotations and memos are made for striking observations*

*Small silences (0-2s) or not separately coded, because they are perceived as the natural silences in a conversation.*

*CODES for COMMUNICATION BEHAVIORS & OTHER OBSERVATIONS*

*The following codes are for other communication behaviors observed in team and patient-student communication in the VR simulations.*

| **COMMUNICATION BEHAVIORS** | |
| --- | --- |
| Code 26 | |
| Label | **Suggestive questioning** |
| Definition | Asking a suggestive question, where one suggests the answer that is expected to follow. The answer already lies within the question. This is a non-preferred ‘skill’. |
| Description/**excerpt** | M15: You're also getting a little worked up, aren't you, sir?  P: Oh yeah. It's such a suffocating, painful feeling, isn't it?  M15: yes. |
| Code 27 | |
| Label | **Bloc of picking up on what patient says** (content) |
| Definition | Students picking up on what patient says with a sequence of different possible skills, f.e. open or closed questioning, paraphrasing, repeating… As long as, content wise, the student is picking up one the patients’ message. Code the premade bloc of orientation. |
| Description/**excerpt** | P: Ooh. What am I going to have? Ooh, but pain.  L11: We are looking, sir, what is wrong.  P: Ooh. But I am so worried.  I21: And what are you worried about, sir?  P: Yes, that it is going to be cancer, right? Ooh. (3.0)  L11: Turn on your blood pressure monitor too.  P: Yes, come on, that all hurts there on my chest, my lungs are there. That is going to be it, right?  L11: [And-]  I21: [And is it] pain on your chest or more in your heart region or where is the pain mainly located.  P: Mainly in the middle.  I21: In the middle. |
| Code 28 |  |
| Label | **Interruption by a student** |
| Definition | A student interrupts another student or the patient, which may cause the other person to stop speaking. *(When – is used within an utterance, it will not be coded, because it is the student changing words withing an utterance.)* |
| Description/**excerpt** | P: [Ooh.] But can I have something for the pain?  E22: We're going to, uhm, yes, quite-  N12: Yes, we can still give you something for that  E22: An IV, [that] is hanging by your side. |
| Code 29 |  |
| Label | **Continuing immediately, leaving no pause/silence** |
| Definition | There is no pause and no interruption. Speaking continues immediately. **(part or bloc)** |
| Description/**excerpt** | A11: =Uhm, otherwise we'll start a full investigation sometime? Uhm. What do you think of an ABCDE?  V21: Yes, definitely.=  A11: =Okay. Yes, the A is already good anyway. Maybe we'll hook up the oxygen saturation meter, uhm? |
| Code 30 | |
| Label | **Two or more people talking at the same time: 1 overlap** |
| Definition | **Initiated by student** (*not when patient initiates this*). Talking is **more** than a vocalization (f.e. ‘hmmm’, ‘oh’), word (f.e. ‘Ja’, ‘nee’, ‘oké’) or one other utterance at the same time. It is a single overlap. |
| Description/**excerpt** | L11: Uhm yeah, but, it's just a thermometer and a syringe here  (7.0)  P: Ooh, are you going to [give something for the pain?]  L11: [Yes, we're going to give something for the pain, right] Agreed that we give something for the pain?  I21: Yes, indeed. |
| Code 30a | |
| Label | **Bloc of two or more people talking at the same time: continuing overlap** |
| Definition | **Initiated by student** (*not when patient initiates this*). Talking is **more** than a vocalization (f.e. ‘hmmm’, ‘oh’), word (f.e. ‘Ja’, ‘nee’, ‘oké’) or one other utterance at the same time. |
| Description/**excerpt** | A11: Uhm, then we'll look at the parameters again. Yes, the saturation is okay, the blood pressure has dropped a bit now. The rhythm is still sinusoidal. [( )]  V21: [Heart rate] is still 105.=  A11: =Yes, which [is normal] for the pain.  V21: °[Saturation is 97]° Yes, that's possible [°( )°]-  A11: [Maybe] we'll look at the parameters again, uhm, °I'm thinking, yes, we don't see signs of meningitis directly with the D either, I think° [Uhm.]  V21: [°wait, hey°] |
| Code 31 | |
| Label | **Students speaking next to each other, not picking up on fellow student (Speaking in parallel)** |
| Definition | Responding (or not) to each other’s questions or utterances with irrelevant information not picking up onto each other (f.e. responding with a not related question or subject, like following own thinking process/agenda). They don’t speak about a common subject. |
| Description/**excerpt** | R13: So here's that heartbeat, then I can (2.0)  M23: °I'm looking for the blood pressure monitor. ( )° (7.5)  R13: Ah, I can't do that. (9.0)  M23: °Wait, is there something here? Saturation ( )° (3.0) Is that even connected? °Oxygen mask or blood pressure?° (7.5) °( ) (2.5) next to the mask ( )° (2.5) |
| Code 32 | |
| Label | **Students not picking up on patient utterances** (ignoring) |
| Definition | Continuing to talk with fellow student, even though the patient is asking thing or speaking in phrases to the students. It is almost like **ignoring**. Vocalizations are not allowed for this code. |
| Description/**excerpt** | V12: I can't do some things. But I can't °get any closer.° (3.5)  P: Ooh, help me. What's wrong? Ooh, that pressure. Ooh.  D: Step back a bit, ( ) (2.0)  P: Ooh, that pressure on my chest, it's only getting worse.  S22: So the capillary refill was normal, right, [you say?]  V12: [Yes, it says] that it's normal. (2.5)  S22: ( ) Maybe the blood pressure again? ( )  V12: Yes, press it again. Yes, °it's about the same now, okay.° Uhm.  P: Ooh, what's wrong with me? That pressure, that pressure. Aah.  V12: ( ) °with circulation.° (3.0) |
| Code 33 | |
| Label | **No initiation of patient-contact at scenario start** |
| Definition | At the start of the scenario, students don’t make contact with patient. They speak ‘above’ the patient or with each other. |
| Description/**excerpt** | A27: Airway first?  L17: What?  A27: °Are we going to check airway, airway° first, or? [( )]  L17: [( )]  A27: Yes, wait. (6.5) |
| Code 34 | |
| Label | **Responding to a patients’ emotions or concerns with a generic reaction** |
| Definition | The answer is **not authentic** in nature, suggested by paraverbal behaviors (f.e. speeding up or immediately continuing an(other) actions; also noted between brackets and smaller text) or verbal behavior (f.e. ’stereotype’ or ‘generic’ phrases or sentences). The student is not picking up on patient. No empathic, affirmational or supportive statement. **(part)** In doubt, better to score then not to score. |
| Description/**excerpt** | P: Oh, and I'm so afraid now that it's going to be lung cancer, right?  M15: But sir, we're going to examine you first, right? Then we'll be able to tell you better what you have.  P: Yes. I'm afraid that's what it's going to be, you know.  M15: I WOULD NOT IMMEDIATELY ASSUME THE WORST.  P: Yes, if it is, it's my own fault, right? |
| Code 35 | |
| Label | **Patient mentions emotions or concerns** |
| Definition | Not about a physical complaint such as pain (f.e. “dat doet pijn”, “het gaat niet”). When there is a hidden concern in a question or utterance, it can be scored. In doubt, better to score then not to score. |
| Description/**excerpt** | P: Ooh. That's not going to be cancer, is it?  E22: Are you worried about that, Danny?  P: Yes, yes yes  E22: And is there anyone in the family who has that, or?  P: Ah yes, my brother.  E22: Yes.  P: And he had that too, those complaints that he was like that ( ). But yes. Ooh.  E22: And tell me, how did you get up this morning? |
| Code 36 | |
| Label | **Not verbally confirming the received information or tasks** |
| Definition | Receiving the message of a fellow student is not verbally or non-verbally acknowledged. Understanding therefore is not clear. Clear behaviors confirming the **closed loop communication** (f.e. yes, oké, picking up on information ) |
| Description/**excerpt** | A11: =Maybe we'll go to twelve sometime, ah yeah ( ), so the rhythm is okay.  V21: °I'm looking at° the [blood pressure too, maybe]  A11: [Then], the saturation, how is that at the moment?=  V21: =92. |
| Code 37 | |
| Label | **Asking multiple questions in one utterance** |
| Definition | The student asks more than 1 question in one utterance. Therefore the patient is likely to answer just one of them (but not always).   - Always code the questions AND the answer (the next utterance). |
| Description/**excerpt** | E22: Hi Danny, how are you? Tell me, what happened?  P: Yeah, how are you. Not good, huh? Oh, I really don't feel good. |
| Code 38 | |
| Label | **Communicating physical touch to patient in examinations** |
| Definition | Student communicates with patient when they will be touching the patient for a physical examination. This is not the same as the context when communicating physical examination to the fellow student, which is team oriented communication. |
| Description/**excerpt** | R14: =I'll put my hands on your chest sometime, hey, if that's okay with you, sir.  P: Yes, yes. Do whatever it takes. (5.0) |
| **Code 39: Engagement in patient communication** | |
| Code **39a** | |
| Label | **A bloc of both students being engaged in patient oriented communication** |
| Description | Both students are engaged in actively listening and speaking with the patient, by picking up on what patient says, by asking questions, by reassuring… Both students connect, interact with patient. |
| Description/**excerpt** | P: Ooh. But pain, pain, pain that I have on my chest.  E22: Pain in your chest. And a pressing pain, or?  P: Yes, yes. It's really pressing.  E22: Yes, well, we're looking now for an ECG. °Did you get the last one, (first name N12)?  N12: Yes, or that there's something wrong with your heart?  E22: And then we can look at the [activity of your heart].  N12: [voila] |
| Code **39b** | |
| Label | **A bloc of one student being engaged in patient oriented communication** |
| Description | Only one student is engaged in actively listening and speaking with the patient, by picking up on what patient says, by asking questions, by reassuring… Both students connect, interact with patient. |
| Description/**excerpt** | B19: Do you have COPD, sir?  P: What?  B19: Do you have something wrong with your lungs? A condition?  P: No [no]  B19: [COPD] they sometimes say  P: No, not that I know of. But I don't have lung cancer, do I?  B19: No, no, no.  P: Ooh. |
| Code 40 | |
| Label | **Verbal acknowledgment of a received nudge** |
| Description | Speaking out loud one received a nudge and/or what the nudge is (content). |
| Description/**excerpt** | E29: No, I can't see anything.  P: Ooh, my chest hurts so much. Ooh.  B19: I'm getting a message. Grab the purple boxes. (7.5) |
