# Supplementary material for: Training communication skills in a multiuser medical virtual reality simulation: a qualitative, observational study
Source: Adv Simul (Lond). 2025 Nov 24;10:59. doi: 10.1186/s41077-025-00386-8 (PMC12642034; doi:10.1186/s41077-025-00386-8)
Supplement: Supplementary file 3 — Supplementary Material 3: Appendix 3. Demographics of participants, collected through qualitative surveys. [file 41077_2025_386_MOESM3_ESM.docx]

**Appendix 4**

Demographics of participants, collected through qualitative surveys.

| **Demographics** | | | |
| --- | --- | --- | --- |
| **Students** | | | **Numbers** |
| Educational level | | *3th bachelor in Medicine* | / |
| Students (identifying as) | | *male* | 9 |
|  |  | *female* | 13 |
| Participation pretraining | | *Three weeks in advance (15 minutes)* | 16 |
|  |  | *At the start of the simulation (5 minutes)* | 6 |
| Non-VR simulation experience (participation) | | *peer* | 14 |
|  |  | *patient* | 20 |
|  |  | *mannequin* | 20 |
| VR-experience (non-educational context) | | *None* | 10 |
|  |  | *Played at least 1 VR-game* | 12 |
| **Teachers** | | | **Characteristics** |
| Facilitator-teacher | *Identifying as* | | female |
|  | *Clinical experience* | | - Nursing - 10 years |
|  | *Non-VR simulation experience (facilitation)* | | - 5 years - Up to 60 simulations |
|  | *VR simulation experience (facilitation)* | | - Within development phase of Smart Collaboration Tutor |
|  |  | |  |
